# Immunoglobulin and Monoclonal Antibody Therapies in Guillain-Barré Syndrome

**DOI:** 10.1007/s13311-022-01253-4

**Published:** 2022-06-01

**Authors:** Yusuf A. Rajabally

**Affiliations:** 1 Aston Medical School, Aston University, Birmingham, B4 7ET UK; 2 Inflammatory Neuropathy Clinic, University Hospitals Birmingham, Queen Elizabeth Hospital, Birmingham, B15 2TH UK

**Keywords:** ANX005, Eculizumab, Guillain-Barré syndrome, Immunoglobulin, Monoclonal antibody, Plasma exchange

## Abstract

Guillain-Barré syndrome (GBS) is an acute autoimmune polyradiculoneuropathy affecting 1–2 subjects per 100,000 every year worldwide. It causes, in its classic form, symmetric weakness in the proximal and distal limb muscles with common involvement of the cranial nerves, particularly facial weakness. Respiratory function is compromised in a case in four. Randomised controlled trials have demonstrated the benefit of therapeutic plasma exchange in hastening time to recovery. Intravenous immunoglobulin was subsequently shown to be as efficacious as plasma exchange in adult subjects. In children, few trials have shown the benefit of intravenous immunoglobulin versus supportive care. Pharmacokinetic studies suggested a relationship between increase in immunoglobulin G level post-infusion and outcome, implying administration of larger doses may be beneficial in subjects with poor prognosis. However, a subsequent trial of a second dose of immunoglobulin in such subjects failed to show improved outcome, while demonstrating a higher risk of thromboembolic side-effects. Monoclonal antibody therapy has more recently been investigated for GBS, after multiple studies in animal models, with different agents and variable postulated mechanisms of action. Eculizumab, a humanised monoclonal antibody against the complement protein C5, was tested in in two randomised, double-blind, placebo-controlled phase 2 trials. Neither showed benefit versus immunoglobulins alone on disability level at 4 weeks, although one study importantly suggested possible, clinically highly relevant, late effects on normalising function. A phase 3 trial is in progress. Preliminary results of a placebo-controlled ongoing study of ANX005, a humanised recombinant antibody against C1q inhibiting the complement cascade, have been promising.

**Supplementary Information:**

The online version contains supplementary material available at 10.1007/s13311-022-01253-4.

## Introduction

Guillain-Barré syndrome (GBS) is a common acute polyradiculoneuropathy, first described by Guillain, Barré and Strohl in 1916 [1]. Its reported incidence varies between 0.8 and 1.9 per 100,000 per year worldwide [2], increasing with age, and the disease is commoner in males [3]. A preceding infection can be identified in about 70% of cases, and GBS represents a model for post-infectious auto-immune disorders [4]. The most common preceding infection causing GBS has been shown to be *Campylobacter jejuni* enteritis, responsible in up to 50% of cases [5, 6]. Other incriminated infectious agents include CMV (cytomegalovirus), EBV (Epstein-Barr virus), *Mycolasma pneumoniae*, *Haemophilus influenzae*, and more recently, Hepatitis E and Zika virus [4, 7–10]. SARS-CoV2 has also been shown to cause GBS in multiple studies from different countries [11–13]. In its classic form, GBS causes acute and rapidly progressive diffuse proximal and distal weakness of the four limbs, sensory symptoms, with often minimal sensory loss, and areflexia [14, 15]. Although, by definition, maximal weakness is reached within 4 weeks, nadir is frequently attained within 2 weeks [16]. Facial and bulbar weakness is common, and autonomic features are well described. Respiratory muscle weakness occurs in 25% of cases, requiring ventilatory support, and represents the main reason why GBS is a life-threatening illness [14].

The latest classification of GBS and the pathophysiologically related Miller Fisher syndrome (MFS) has provided descriptive clarification of the variable clinical presentations of these 2 entities [17]. Several variants of GBS have been reported. Besides the classic form, focal forms of GBS include the paraparetic variant [18], the pharyngocervicobrachial (PCB) variant [19], the variant with acute (pure) pharyngeal or bulbar weakness [20] and bifacial weakness with (or without) distal paraesthesiae [21]. Classic MFS is characterised by the triad of ophthalmoplegia, ataxia and areflexia [22]. MFS can co-exist with classic GBS, in which motor weakness is additionally present. Other forms of MFS include acute (isolated) ophthalmoparesis, acute (pure) ataxic neuropathy, acute (isolated) ptosis, acute (isolated) mydrasis, Bickerstaff’s brainstem encephalitis (BBE) which involves central nervous system involvement, producing somnolence as well as eye movement disorders, and acute ataxic hypersomnolence [23], which represents an incomplete form of BBE without ophthalmoparesis.

The diagnosis of GBS is clinical, which may be aided by electrophysiology although this is not essential and may be found normal in early disease. In broad terms, electrophysiology separates demyelinating forms, categorised as “acute inflammatory demyelinating polyradiculoneuropathy”, or “AIDP”, or axonal forms, which have been described variably, on purely electrophysiological grounds, as “acute motor axonal neuropathy” or “AMAN”, “acute motor and sensory axonal neuropathy”, or “AMSAN” and “acute motor conduction block neuropathy”, or “AMCBN” [24, 25]. The methods for electrophysiological classification are variable, have been the subject of debate [26], but are beyond the scope of this article. It is important to note that this classification brings insight into the site of the immune attack but does not have any implications for treatment modalities. Hence, the value of electrophysiology, besides adding support to the clinical diagnosis, is low. A rise in cerebrospinal fluid (CSF) protein level with normal CSF cellularity, also known as “albumino-cytological dissociation”, is characteristic of GBS, but similarly, although present in over 90% of patients 2 weeks post-onset, may be normal, particularly in early disease stages [27].

**Fig. 1:**
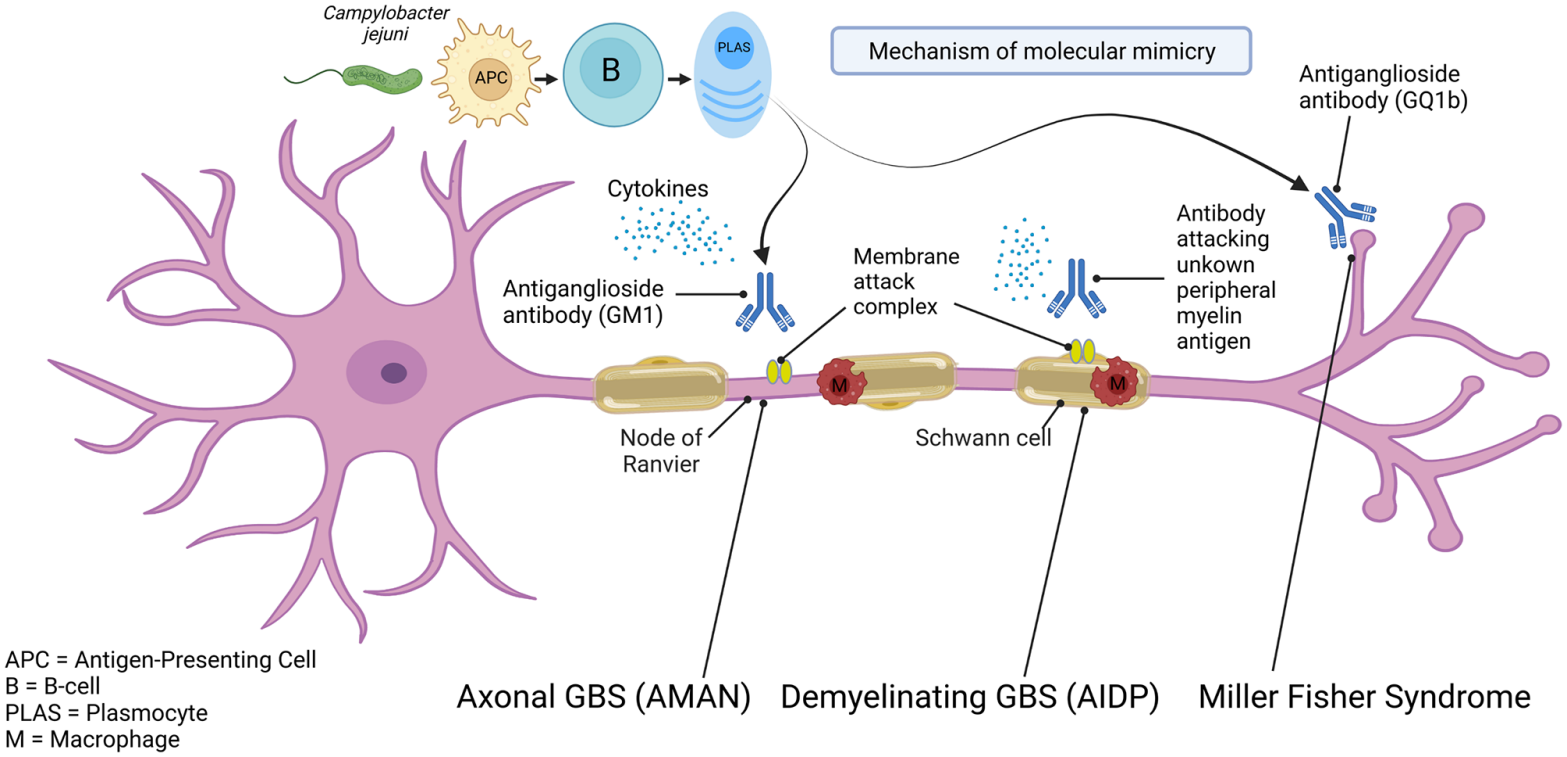
Mechanism involved in the pathogenesis of GBS and MFS

Pathophysiologically, evidence points towards a humorally mediated process in both AIDP and axonal forms of GBS, as well as MFS [28]. This is summarised in Fig. 1. The immune attack is directed towards myelin components in the former and the node of Ranvier, the paranodal and juxtaparanodal regions, in the latter. Molecular mimicry between microbial antigens and axolemmal components represents the basis of the immune process in axonal forms, with preceding infection to *Campylobacter jejuni* causing production of anti-lipo-oligosaccharide antibodies which then bind to identical nerve gangliosides [29]. These antiganglioside antibodies, mainly directed towards GM1 and GD1a in axonal GBS and towards GQ1b in MFS, cause axonal injury at nodal regions and nerve terminals, resulting in conduction block, which may itself be reversible, with subsequent good clinical recovery, or alternatively, be followed by axonal degeneration and poor clinical recovery [30, 31]. The underlying basis of this difference in outcome is unknown. In addition to antibody-mediated attack, complement activation contributes to the pathological process in disrupting sodium channel clusters at the nodes of Ranvier [32], and the activation of dendritic cells by *Campylobacter jejuni* lipo-oligosaccharides induce B-cell proliferation through interferon 1 and tumour necrosis factor production [33]. In demyelinating forms, mechanisms are poorly understood at the present time, with a range of antibodies directed against myelin structures, being possibly involved [28]. As regards therapeutic implications, there are currently no practical treatment differences between the various GBS subtypes, for which only plasma exchanges (PE) and intravenous immunoglobulin have been found of benefit.

**Fig. 2:**
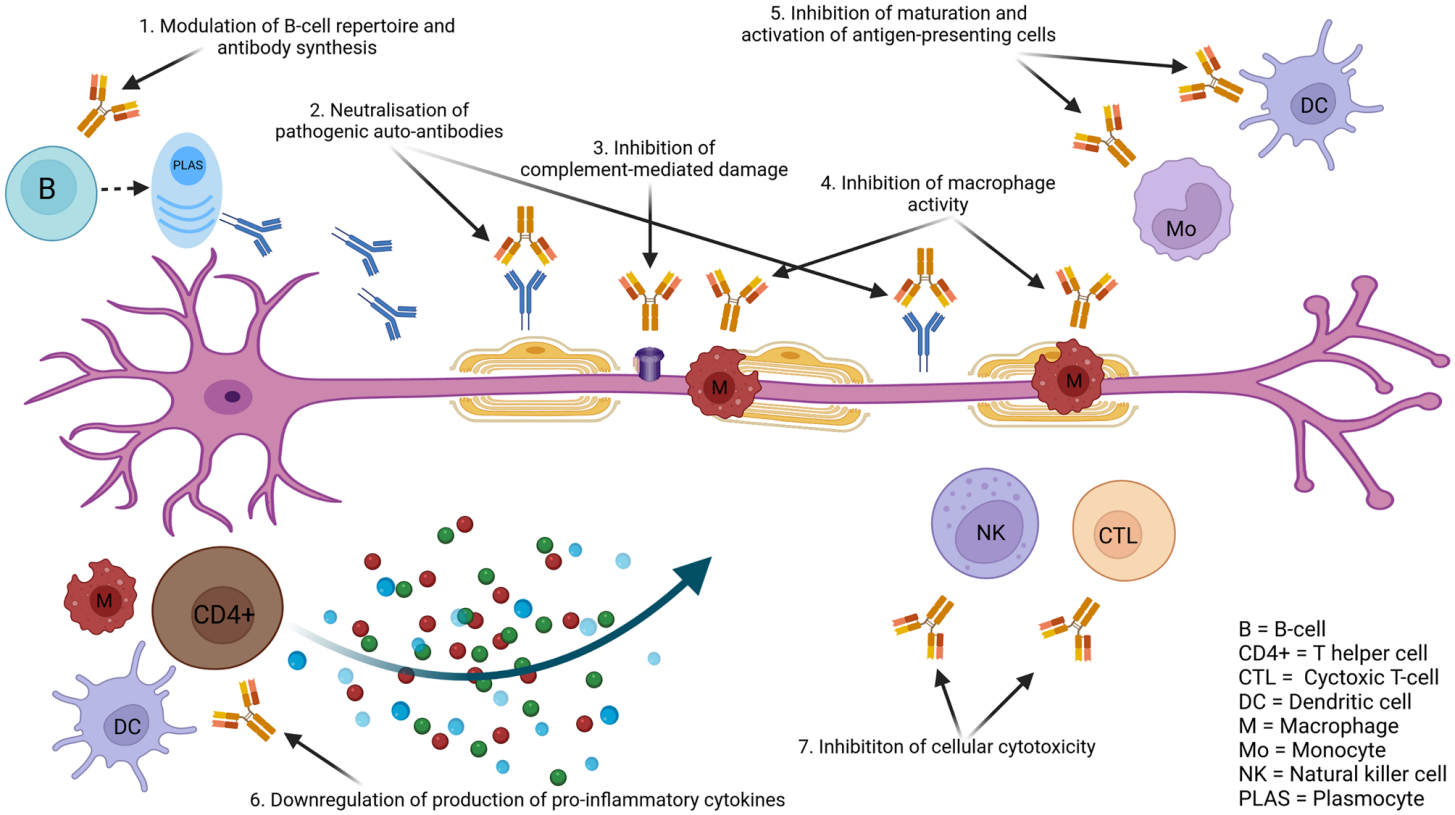
Postulated mechanisms of action of immunoglobulin therapy in GBS

The efficacy of PE has been shown in the treatment of GBS through several trials. The therapeutic use of PE in GBS is theoretically justified by the need to remove neuro-toxic inflammatory agents that cause the condition following preceding infection, and mechanisms of molecular mimicry, established in axonal forms, as described above. Immunoglobulin therapy was later found to be of comparable efficacy in head-to-head comparative studies with PE. The mechanisms through which immunoglobulin therapy may be effective in GBS are potentially multiple and relate to the different pathophysiological mechanisms implicated in the active phase of the disease [34]. They include blockade of Fc receptors on macrophages preventing macrophage-induced damage, neutralisation of pathogenic antibodies, anti-cytokine activity, inhibition of antibody production and increase of antibody catabolism, complement inhibition, as well as regulatory T-cell effects [35]. These potential mechanisms of action of immunoglobulin treatment in GBS are summarised in Fig. 2. Prompt administration of PE or immunoglobulin treatment is advisable to rapidly act upon the early stages of the peripheral nerve-directed immune attack, with greater likelihood of prevention of irreversible damage and, as a result, better prognosis.

## Evidence Base for Immunoglobulin Treatment in GBS

The treatment of GBS remains today based on results of trials conducted over three decades ago. Initial research demonstrated the efficacy of PE. Subsequently, studies comparing PE and immunoglobulins showed equivalence of these 2 treatments. There is otherwise no evidence for use of other tested agents for GBS, including steroids [36], and interferon-beta 1a [37] as well, to date, of monoclonal antibody therapy (see section below). This led to immunoglobulin becoming the first-line therapy essentially due to ease of administration. This situation has, in recent times, been reversed due to reduced immunoglobulin availability in some parts of the world, including the UK, leading to increased usage of PE. To date, PE and immunoglobulins represent the only 2 evidence-based treatments available for GBS. The current evidence base supports treatment in cases where patients are unable to walk 10 m independently. There is limited evidence for milder forms of GBS. Two rather than 4 sessions of PE have been found beneficial in mild GBS [38], although no comparative data for reduced doses of intravenous immunoglobulins are available. In clinical practice, treatment is considered in patients able to walk independently, in case of bulbar or respiratory involvement, when dysautonomia occurs, when there is disabling upper limb weakness, or in the presence of rapid decline [39].

The first randomised trial for GBS evaluated PE versus supportive treatment alone and was published in 1984. This, probably underpowered, study was negative for the primary outcome measure, which was functional ability at 4 weeks [40]. Several further trials were subsequently performed, demonstrating short-term benefit, as well as improved long-term recovery [41–44].

The latest version of the Cochrane review on the evidence for PE in GBS from 2017 detailed the effects of the treatment by sub-analysis of the different outcome measures utilised in the different trials [45]. A grand total of 649 patients were included in this meta-analysis, with variable numbers analysed for the different studied outcome measures. The review used as primary end-point the time to recover walking with aid (Hughes disability grade 3). The meta-analysis demonstrated that this time was significantly shortened in both severely affected and mildly affected PE-treated GBS patients (30 days versus 44 days and 6 days versus 10 days, respectively). Other outcomes which were favourable with PE included time to onset of motor recovery (6 versus 10 days; *p* < 0.0001), mean improvement in Hughes disability grade at 4 weeks (RR: 1.6; 95% CI: 1.19–2.15), time to recover walking without aid (RR: 1.72; 95% CI: 1.06–2.79), need for mechanical ventilation (RR: 0.53; 95% CI: 0.39–0.74), likelihood of full motor strength recovery at 1 year (RR: 1.24; 95% CI: 1.07–1.45), severe motor sequelae at 1 year (RR: 0.65; 95% CI: 0.44–0.96). On the other hand, PE did not reduce risk of death at 1 year (RR: 0.86; 95% CI: 0.45–1.65) and appeared more commonly associated with GBS relapse (RR: 2.89; 95% CI: 1.05–7.93). Otherwise, although more efficacious when administered in the first 7 days, PE was also effective when offered between 7 and 30 days after disease onset, for most outcome measures. Finally, there was no difference in rates of occurrence of side-effects, also considering blood pressure instability, cardiac dysrythmia or pulmonary embolism, between the PE-treated and control groups.

There have been no trials of immunoglobulin versus placebo or supportive care only in adults with GBS. Immunoglobulins have been studied in GBS in several comparative studies with PE, which included over 500 adults in total. No significant differences were observed considering change in disability level at 4 weeks. Immunoglobulin treatment was effective when administered within 2 weeks of onset of weakness, with no established evidence of benefit after this window.

In different trials of adult subjects, performed between 1992 and 2001, only the first, which was unblinded, showed significant superiority of immunoglobulin over PE at 4 weeks [46]. All others [47–49], including the largest, of over 250 participants receiving either of the 2 treatments, which was the only blinded study [47], showed no significant differences.

One unblinded paediatric study demonstrated superiority of PE over immunoglobulin in ventilated children, in improving duration of respiratory support, but not duration of intensive care stay or functional neurological outcome at 4 weeks [50].

The only trials of immunoglobulin versus supportive care only were performed in children [51, 52]. Mean improvement on the GBS disability scale and rate of recovery of full strength at 4 weeks were greater in immunoglobulin-treated patients. One immunoglobulin dose-comparative study (400 mg/kg daily given for 3 days versus, for 6 days) showed a trend for greater benefit in the prolonged treatment course in terms of median time taken to be able to walk with aid, but this result did not reach statistical significance [53]. Another study compared the duration of dose-equivalent immunoglobulin therapy in children (400 mg/kg daily for 5 days vs. 1.0 g/kg daily for 2 days) [52]. No significant differences in outcome were found, although interestingly, relapses occurred significantly more commonly with the shorter regimen.

One of the above-mentioned trials compared immunoglobulin vs. PE followed by immunoglobulins [47]. No significant difference of outcome could be ascertained at 4 weeks. Another analysis evaluated immunoglobulin alone in comparison to immunoglobulin with pulse corticosteroid therapy [54]. No additional benefit of corticosteroids could be demonstrated, in keeping with data from other studies which showed no effect of corticosteroids alone [36].

In summary, and as illustrated by the relevant Cochrane review [55], no differences were found on meta-analysis of the different trials, between PE and immunoglobulin for functional outcome at 4 weeks, time to discontinuation of mechanical ventilation, death or disability at 12 months. It is however noteworthy that non-randomised studies otherwise suggested that subjects with axonal GBS harbouring antiganglioside antibodies recovered quicker after immunoglobulin treatment instead of PE [56–58]. These interesting as highly relevant findings for clinical practice have unfortunately not been adequately further studied since. Importantly, despite a trend in favour of immunoglobulin, no significant differences in the rate of occurrence of side-effects was found comparing the 2 treatments, the findings limited however by non-uniformity between different trials for classifying adverse effects and causality [55].

## Immunoglobulin Treatment of MFS and BBE

The available evidence for treatment of acute polyradiculoneuropathies relates to patients meeting definitions for GBS, with progressive weakness of 2 or more limbs over a maximum of 4 weeks, hypo- or areflexia, absence of an alternative cause and resulting inability to walk independently. There is no randomised controlled trial data for MFS. The close relationship between MFS and GBS as well as anecdotal reports of the benefit of PE and immunoglobulin for patients with MFS however led to widespread use of immunomodulation in clinical practice. A first retrospective Japanese study of 50 patients with MFS showed no difference between PE and no treatment, for time required to resolve, or chances of recovery from, ataxia and ophthalmoplegia [59]. Another retrospective study from the same Japanese group of 92 patients with MFS, comparing immunoglobulin, PE and no treatment, showed no influence of intervention on ultimate outcome, considering time of disappearance of ophthalmoplegia or ataxia, and presence of residual symptoms at 1 year [60]. Immunoglobulin however appeared to hasten the start of improvement of ophthalmoplegia and ataxia in comparison to no treatment, no differences being found comparing immunoglobulin and PE as well as PE and no treatment. Considering the nature and potential subjective patient as well as examiner bias of the assessment methods used, it appears difficult to conclude in any definite benefit of immunoglobulin therapy, particularly given the sample size studied and the retrospective, non-randomised design.

The treatment of BBE has similarly not been investigated [61]. As a result of its clinical severity and known mortality rate, both PE and immunoglobulin are however often used by clinicians. There is however no evidence for this, and with regard to immunoglobulin, careful consideration of the risk of thromboembolic complications is needed, considering most patients with BBE recover fully or almost fully, with minimal residual symptoms, within 6 months [61]. Hence, that BBE justifies treatment because of its severity [62] is debatable. In any MFS variant, including BBE, overlap with GBS is however, on the other hand, a justification for treatment as isolated GBS.

## The Pharmacokinetic Hypothesis of Variable Immunoglobulin Effectiveness in GBS

Despite the above-mentioned trials demonstrating a favourable effect of immunoglobulin on outcome in GBS, it is clear that the treatment may unfortunately have little or no effect in a proportion of patients, as illustrated by study of described outcomes of treated patients from trials [63]. Prognostic models have shown several features, including greater age, diarrhoea and greater deficits in the early stage, admission delay, facial and bulbar weakness, as associated with poor outcome [64–66]. The role of immunoglobulin pharmacokinetics as potential contributory factor to prognostic variability in GBS was rightly considered as an avenue to investigate.

In 2009, Kuitwaard et al. reported on a study of 174 Dutch patients with GBS, who had previously participated in 2 therapeutic trials with immunoglobulin [67]. Evaluating the immunoglobulin G (IgG) level pre- and post-treatment at 14 days, they found that the increase in serum IgG (ΔIgG) was very variable 2 weeks after infusions at a standard dose of 2 g/kg of body weight, with a better outcome at 6 months being independently associated with higher ΔIgG levels. This analysis found that quartiles of subjects grouped per increase in ΔIgG level had significantly different outcomes at 6 months, varying from 28% with bad outcome defined by a GBS severity score > 2 in the group with the lowest increase (< 3.99 g increase), to only 7%, in that with the highest increase (> 10.92 g). Combining these findings with clinical experience and data from case series which suggested possible efficacy re-treatment with immunoglobulin in GBS led to consideration of the possibility that some individuals may have a quicker IgG clearance and hence require higher dose or repeat treatment, to achieve a potentially improved outcome. However, the data from that initial study, despite the described conclusions of association of outcome at 6 months with ΔIgG levels, raised some interesting but concerning questions. Notably, with regard to the baseline characteristics of clinical severity at entry, using both GBS disability scores and MRC scores, associations were found with, the only subsequently measured, ΔIgG levels at 2 weeks. The authors hypothesised, in this regard, that a higher degree of neuroinflammatory damage may result in a greater consumption of IgG. They also postulated that more affected patients may be affected by a higher rate of infections acquired in intensive care, which may increase IgG catabolism as has been found to occur in sepsis and severe trauma requiring mechanical ventilation. The other factor they mentioned as possible explanation for smaller increase in ΔIgG levels was a high baseline serum IgG. However, this was not clearly substantiated by the data provided in their study. Despite, in this context, the possibility that more severe disease may result in greater IgG catabolic rate, the more optimistic hypothesis of the latter resulting in poorer outcome was considered, implying the possibility of greater efficacy of administration of higher immunoglobulin doses.

This hypothesis that increasing ΔIgG levels through increasing the dose of immunoglobulin administration may ultimately produce better clinical outcomes in patients with severe GBS was tested in a double-blind, randomised, placebo-controlled trial performed over an extended period of > 8 years (2010–2018), the SID-GBS (“Second intravenous immunoglobulin dose in patients with GBS with poor prognosis”) [68]. In the interim, analysis of a non-randomised study of the International GBS Outcome Study (IGOS), greatly limited by small numbers and suboptimal design, found no beneficial effect of a second dose immunoglobulin treatment [69]. The results of the SID-GBS study were themselves published in 2021, over a decade after the start of the trial [70]. This multicentre study performed in 59 institutions in the Netherlands included a total of 327 subjects aged ≥ 12 years. All were treated with on admission with standard intravenous immunoglobulin at 2 g/kg over 5 days. Of those, 96 patients, who had a poor prognostic score (≥ 6), as per the modified Erasmus GBS Outcome Score, received either a similar second dose of immunoglobulins, or placebo, 7–9 days after inclusion. The primary outcome was the GBS disability score 4 weeks after inclusion. After adjustment for known prognostic factors, for which the distribution within the 2 intervention groups was uneven, no difference could be found for the primary outcome, in favour of a second immunoglobulin dose (O.R.: 1.4; 95% CI: 0.6–3.3). Similarly, no difference could be found for any of the secondary outcomes, including at 8, 12 and 26 weeks, using multiple measures including GBS score, Medical Research Council strength (MRC) sum score, Overall Neuropathy Limitation Score, duration of hospital stay, duration of intensive care treatment and of mechanical ventilation. In contrast, the risk of severe adverse events, including thromboembolic, was significantly higher in patients treated with a second immunoglobulin dose (O.R.: 3.54; 95% CI: 1.44–8.72).

Despite the confirmed increased IgG levels in patients treated with a second dose, this study therefore showed no concurrent improved outcome. This had been a potential sole conclusion of the initial pharmacokinetic study, in keeping with reported high IgG consumption in post-surgical infection [71], and indicated low ΔIgG levels as a consequence rather than as a cause of severe GBS. The limitations as well as hazards of repeat immunoglobulin treatment have now, importantly, through the results of the SID-GBS study, become evident as has the need for immediate change of clinical practice, which involves over a third of unresponsive patients being re-treated [72].

## Immunoglobulin Treatment of GBS: Future Perspectives

The efficacy of immunoglobulins in all forms of GBS is well-demonstrated in case of sufficient clinical severity, when administered within the first 2 weeks after weakness onset. Immunoglobulins remain in general easier to administer and more accessible in most parts of the world, and the issue of cost and relative unavailability must be counterbalanced by awareness of the low incidence rate of GBS and of its monophasic nature, as well as the recently shown inappropriateness of repeat treatment.

Decisions for immunoglobulin treatment versus PE should, in practice, mainly reflect consideration of individual patient risk factors, particularly thromboembolic [73, 74]. The treatment decision requires careful clinical evaluation and knowledge of the existing evidence in relation to the generally favourable natural history of GBS, as well as the high accuracy of known predictors of poor prognosis from onset [65]. Similarly, symptom severity in MFS and BBE should be remembered as not inconsistent with excellent subsequent spontaneous recovery, rather than a justification for treatment, for which there is no evidence. Dosing of immunoglobulins is a rarely discussed topic generally in treatment of autoimmune neurological disease, even more so for the one-off infusions offered to patients with GBS. The empirical dose of 2 g/kg may however clearly be inappropriately high in subjects with high BMI, and use of ideal body weight, or dosing weight formulae, as routinely done in the UK, may be an effective way of reducing side-effect risk as well as costs [75].

The recent SID-GBS trial has been invaluable in demonstrating the absence of justification of repeat immunoglobulin courses in severe GBS, a practice which has been widespread until now, despite previously demonstrated absence of effect of enhanced or combined immunotherapies. Furthermore, the increased vascular risk in re-treated patients raises the issue of whether this is justified in case of treatment-related fluctuations, for which evidence of benefit of repeat infusions vs. no further treatment, is unproven.

The high cost and low availability of immunoglobulins unfortunately means that most patients affected by GBS worldwide remain untreated. PE is not a much cheaper option. Mini-pool immunoglobulins, collected from a small number of plasma samples, coming from 20 instead of 3000–10,000 donors for standard immunoglobulins, are quicker and cheaper to produce [76]. They have been evaluated for safety and efficacy in a pilot study in immune thrombocytopenia and encouragingly showed similar outcomes to standard immunoglobulins [77]. A randomised study of 50 participants comparing mini-pool immunoglobulins and PE is now planned in subjects with GBS in Egypt (NCT04550611). If successful, the results may have major positive effects on the care of the many affected subjects living in resource-limited countries, similar to that of small volume plasma exchange, studied in Bangladesh in recent years [78].

## Monoclonal Antibody Therapy for GBS

The absence of useful additional treatment options is highlighted in patients with GBS of unfavourable prognosis and raises the need for research into more effective therapies.

Monoclonal antibodies (mAbs) have become a treatment option in other autoimmune neurological conditions, and their potential in GBS has also been under consideration in recent years. mAbs are of IgG isotype which bind to the epitope of the target with their Fab antigen-binding region, with resulting specific function-inhibition or intracellular signalling. Through binding of their Fc (fragment crystallizable) region, they may induce cytotoxicity [79]. mAbs have, as a result, heterogeneous antigen targets as well as therapeutic mechanisms. In multiple sclerosis (MS), mAbs have represented a major addition to the therapeutic armamentarium, offering targeted mechanism of action and potency in severe relapsing disease. Illustrating their mechanistic heterogeneity, including in a single disease, processes in mAb effects in MS include leukocyte migration inhibition through the blood brain barrier (natalizumab), antibody-dependent and complement-dependent lymphocyte depletion (alemtuzumab) and targeted depletion of CD-20 expressing B lymphocytes (rituximab, ocrelizumab, ofatumumab and ublituximab) [80]. In Neuromyelitis Optica Spectrum Disorders (NMO-SD), a number of mAbs have shown efficacy in reducing relapse rate. Similarly, mechanisms of action of different mAbs used in NMO-SD are heterogeneous. Anti-CD20 mAbs (rituximab, ublituximab), an anti-CD19 mAb (inebilizumab), anti-IL6R mAbs (tocilizumab, satralizumab) as well as a vascular endothelial growth factor A-directed mAb (bevacizumab) have been studied in NMO-SD [81].

Amongst those mAbs effective in NMO-SD, eculizumab is a humanised monoclonal IgG2 antibody against the complement protein C5, today also licensed for myasthenia gravis and used for paroxysmal nocturnal haemoglobinuria and atypical haemolytic uremic syndrome. Complement activation was initially proposed as trigger of demyelination in AIDP from an autopsy study, hence indicating possible therapeutic effect of complement inhibition [82]. Eculizumab was shown to prevent antiganglioside-mediated neuropathy in a murine model, suggesting protection against complement-induced injury [83]. Eculizumab was subsequently tested in a randomised, double-blind, placebo-controlled trial which eventually included only 8 participants after pre-screening of 28 subjects [84]. The main reason for failure to enter the trial was refusal to participate due to perceived risks of meningitis and infection with eculizumab, concomitant pyrexial illness or past history of meningococcal infection. In the treatment group, 5 subjects received eculizumab for 4 weeks, alongside standard immunoglobulin therapy. The primary outcome, improvement by at least one point on the GBS Disability Scale at 4 weeks, was achieved in 2/5 of the treated group vs. 2/2 in the placebo group, a result unfortunately and predictably inconclusive in view of the sample size. The Japanese multicentre, prospective, randomised, phase II study of Eculizumab for GBS (JET-GBS) was conducted between 2015 and 2016, and recruited 34 patients GBS who were unable to walk, randomised in a 2:1 ratio to receive intravenous immunoglobulin and eculizumab, or intravenous immunoglobulin and placebo, respectively [85]. The results were published in 2018 [86]. The primary outcome was the regained ability to walk 5 m independently at week 4, which was attained by 61% of subjects in the treatment group vs. 45% in the placebo group, failing to reach the pre-defined level for significance. Similarly, secondary outcomes including improvement by one functional grade at 4 or 24 weeks, ability to walk 5 m independently at 24 weeks, time to improvement by one functional grade showed no difference between eculizumab and placebo groups. Of interest however, the ability to run at 24 weeks was achieved 17/23 eculizumab-treated patients vs. only 2/11 placebo-treated patients, a significant difference (*p* = 0.004). Various potential explanations for the negative result of this trial may have been the small sample size, sub-optimal dosing of eculizumab, possible neutralisation by concomitant immunoglobulin administration and/or, alternatively, an inadequately chosen primary outcome, given the unexpected but very interesting favourable treatment effect on long-term full recovery, illustrated by the ability to run at 24 weeks. Eculizumab was well-tolerated except for 2 concerning cases of anaphylaxis and cerebral haemorrhage and cerebral abscess. A phase 3 randomised, multicentre, double-blind, placebo-controlled study of eculizumab in severe GBS is now being conducted in Japan (NCT04752566). Of note, the primary outcome has been altered to the time to first reach a Hughes Functional Disability score of ≤ 1, within a time frame of 24 weeks, although all participants remain on immunoglobulin treatment as per standard of care. The study is due to be completed in October 2022.

ANX005 is a humanised IgG4 recombinant mAb against C1q, inhibiting the complement cascade, which has shown promise in reducing immune cell recruitment and axonal injury on animal model of AMAN [87]. A first, phase Ib open-label, single group study to assess safety and tolerability of ANX005 in association with immunoglobulin was conducted on 14 subjects (NCT04035135). A phase Ib, randomised double-blind, placebo-controlled study is taking place in Bangladesh, planning to recruit 180 patients, who, of note, will receive no associated standard treatment with immunoglobulins or PE (NCT04701164). A phase 2/3 study is to follow. Encouraging preliminary results on an analysis of 23 ANX005-treated and 8 placebo-treated subjects were presented in abstract form regarding MRC sum score improvement at week 1, which correlated with GBS Disability Score and Inflammatory Rasch-built Overall Disability Scale scores at week 8 (Papri et al., Abstract 207, Peripheral Nerve Society Virtual Meeting, 2020). GBS disability score improved by ≥ 3 points in 28% of patients treated versus 0% of those on placebo with additional positive trends on length of ICU stay and duration of mechanical ventilation, as well as earlier decline in neurofilament light chain levels in treated subjects.

Several other mAbs, with varying postulated mechanisms of action, have been considered for GBS, mainly through animal studies. BEC2, an anti-GD3 anti-idiotype monoclonal antibody, showed effectiveness in an animal model of GBS, pre-immunised with GD3-like lipo-oligosaccharides isolated from *Campylobacter jejuni* [88]. This suggested the potential for anti-idiotype mAbs in the disorder, which, to date, has not been investigated in humans. Anti-T-cell mAbs used in acute renal hepatic and cardiac transplant rejections were tried in the form of OKT3, a murine antibody directed against all human T-cells, in 3 patients with GBS, early in their disease course [89]. Despite T3 lymphocyte depletion, disease progression continued. Of concern, aseptic meningitis and reactivation of herpes infection occurred in one subject. WT-1, a mAb to lymphocyte function-associated antigen-1, was studied in another animal model of GBS, demonstrating reduced inflammation and demyelination in treated animals [90]. The anti-CD2 mAb OX34 has also been used in an animal model of GBS [91]. Experimental autoimmune neuritis (EAN) was effectively prevented by OX34, which also reduced disease progression if administered after onset. An effect on T-cell migration across the blood nerve barrier was postulated. The anti-L-selectin monoclonal antibody HRL3, blocking the adhesion molecule L-selectin which facilitates trans-endothelial leucocyte migration, was similarly used in an animal model of GBS [92]. Favourable clinical and pathological effects were observed in treated animals, irrespective of the pre-onset or post-onset administration of HRL3. An anti-IL18 mAb was also investigated in animal model of GBS showing amelioration of clinical and pathological features, which were postulated as due to down regulation of Th1 responses to peripheral myelin antigens and reduction of autoantibody responses [93].

On the other hand, rituximab, an anti-CD20 mAb increasingly used in chronic dysimmune neuropathies, including anti-MAG (myelin-associated glycoprotein) neuropathy, paranodopathies and refractory CIDP (chronic inflammatory demyelinating polyneuropathy), has not been the subject of research in GBS, with one anecdotal case report suggesting benefit [94] and others raising the possibility of rituximab contributing to the onset of GBS [95, 96]. It is noteworthy that paranodopathies may have a GBS-like presentation and benefit from early rituximab therapy if appropriately suspected clinically and serologically confirmed. Similarly, alemtuzumab, an anti-CD52 mAb, which has been used in multiple sclerosis and exceptionally in CIDP [97], has not been studied in GBS. One case report describes benefit [98], while others suggest a role of alemtuzumab in causing GBS [99, 100].

## Other Treatments Currently Under Investigation for GBS in Human Studies

Imlifidase is an antibody-cleaving enzyme, originating from *Streptococcus pyogenes*, that specifically targets IgG and inhibits IgG-mediated immune response, and which is used for the desensitisation treatment of highly sensitised adult kidney transplant patients with a positive crossmatch against an available deceased donor [101]. An open single-arm multicentre study is currently taking place in France, planning to recruit 30 participants with GBS to receive imlifidase and standard immunoglobulins, with a comparison planned with a control group from the IGOS study, receiving immunoglobulins only (NCT03943589).

The investigation of the safety and dosing of CK0801, a cord-blood derived T-regulatory cell product, is currently being planned in open-label, single-arm study on patients with severe GBS (defined as GBS Disability Scale score ≥ 4, unchanged 1 week after immunoglobulin or PE), to commence in November 2022 (NCT03773328). T-regulatory cells play a role in limiting autoimmune responses by modulating innate and adaptative immunity.

## Conclusion

Despite the existing evidence base for GBS, more effective treatments are desirable, arguably principally for the subset of patients with high risk of severe long-term disability. Although long-term mild/moderate symptoms and deficits such as pain, fatigue, difficulties returning to work or usual full physical activities persist in a significant proportion of patients [63], none of these manifestations represent the focus of current research efforts. With regard to novel therapeutic mechanisms with mAbs, the findings of the Japanese Eculizumab study [86] suggest changes in research methodology may be needed, considering that long-term prognosis is undoubtedly of greater clinical relevance than short-term outcomes in GBS. This has adequately been taken into account with regard to the chosen primary outcome measure, in the most recent, currently ongoing phase 3 eculizumab trial (NCT04752566). Given the need to prioritise reversing severe disability, more research targeting specific subgroups of poor prognosis remain justified. However, the relative rarity of GBS may lead to the temptation of inclusion of subjects with relatively milder disease and/or better prognosis, which may be both scientifically inadequate as well as ethically questionable. GBS may justifiably be considered of good functional prognosis in most affected subjects, and it may be as important in future to attempt preventing severe persistent disability in a minority, as it is to try reducing long-term symptoms which affect the majority, including those deemed to have well recovered. The therapeutic avenues for these two separate issues may be dissimilar, complicating the task ahead further. Another important aspect to consider is the effect of standard treatment in new drug trials. The JET-GBS study illustrates this, as high-dose intravenous immunoglobulin therapy may have partly neutralised eculizumab [86]. The ongoing ANX005 trial does not involve standard immunoglobulin or PE, this likely made possible as conducted in Bangladesh, where standard evidence-based treatments are not routinely available. Also requiring careful consideration with regard to new treatments is the comparison with existing therapies in relation to side-effect risk, as well-illustrated by the difficulties with recruitment to the UK eculizumab study and the concerning albeit rare, serious adverse events encountered in the Japanese study. Finally, in view of the duty and priority of reducing world health inequity, greater enthusiasm and further research into the potential of mini-pool immunoglobulins are highly desirable.

## Supplementary Information

Below is the link to the electronic supplementary material.

Supplementary file1 (PDF 465 kb)
